# An Automated Glowworm Swarm Optimization with an Inception-Based Deep Convolutional Neural Network for COVID-19 Diagnosis and Classification

**DOI:** 10.3390/healthcare10040697

**Published:** 2022-04-08

**Authors:** Ibrahim Abunadi, Amani Abdulrahman Albraikan, Jaber S. Alzahrani, Majdy M. Eltahir, Anwer Mustafa Hilal, Mohamed I. Eldesouki, Abdelwahed Motwakel, Ishfaq Yaseen

**Affiliations:** 1 Department of Information Systems, College of Computer and Information Sciences, Prince Sultan University, Riyadh 12435, Saudi Arabia; iabunadi@psu.edu.sa; 2 Department of Computer Sciences, College of Computer and Information Sciences, Princess Nourah Bint Abdulrahman University, Riyadh 11671, Saudi Arabia; aalbraikan@pnu.edu.sa; 3 Department of Industrial Engineering, College of Engineering at Alqunfudah, Umm Al-Qura University, Mecca 24382, Saudi Arabia; jszahrani@uqu.edu.sa; 4 Department of Information Systems, College of Science & Art at Mahayil, King Khalid University, Abha 62529, Saudi Arabia; meltahr@kku.edu.sa; 5 Department of Computer and Self Development, Preparatory Year Deanship, Prince Sattam Bin Abdulaziz University, AlKharj 16278, Saudi Arabia; asmaeil@psau.edu.sa (A.M.); iyaseen@psau.edu.sa (I.Y.); 6 Department of Information System, College of Computer Engineering and Sciences, Prince Sattam Bin Abdulaziz University, AlKharj 16278, Saudi Arabia; mldesouki@psau.edu.sa

**Keywords:** deep learning, inception networks, COVID-19, classification, GSO algorithm, radiological images

## Abstract

Recently, the COVID-19 epidemic has had a major impact on day-to-day life of people all over the globe, and it demands various kinds of screening tests to detect the coronavirus. Conversely, the development of deep learning (DL) models combined with radiological images is useful for accurate detection and classification. DL models are full of hyperparameters, and identifying the optimal parameter configuration in such a high dimensional space is not a trivial challenge. Since the procedure of setting the hyperparameters requires expertise and extensive trial and error, metaheuristic algorithms can be employed. With this motivation, this paper presents an automated glowworm swarm optimization (GSO) with an inception-based deep convolutional neural network (IDCNN) for COVID-19 diagnosis and classification, called the GSO-IDCNN model. The presented model involves a Gaussian smoothening filter (GSF) to eradicate the noise that exists from the radiological images. Additionally, the IDCNN-based feature extractor is utilized, which makes use of the Inception v4 model. To further enhance the performance of the IDCNN technique, the hyperparameters are optimally tuned using the GSO algorithm. Lastly, an adaptive neuro-fuzzy classifier (ANFC) is used for classifying the existence of COVID-19. The design of the GSO algorithm with the ANFC model for COVID-19 diagnosis shows the novelty of the work. For experimental validation, a series of simulations were performed on benchmark radiological imaging databases to highlight the superior outcome of the GSO-IDCNN technique. The experimental values pointed out that the GSO-IDCNN methodology has demonstrated a proficient outcome by offering a maximal $sens_{y}$ of 0.9422, $spec_{y}$ of 0.9466, $prec_{n}$ of 0.9494, $acc_{y}$ of 0.9429, and $F1_{score}$ of 0.9394.

## 1. Introduction

The 2019 novel coronavirus named COVID-19 has become a major threat to human health across the globe. Earlier works reported that the Severe Acute Respiratory Syndrome Coronavirus 2 (SARS-CoV-2) began from decomposed cats that affected human beings, and Middle East Respiratory Syndrome (MERS-CoV) virus began from Arabian camels to human beings. It is believed that COVID-19 started in bats and spread to humans. It can infect the respiratory system easily and is rapidly transmitted to other people. It exhibits milder symptoms in about 82% of patients, and the remaining worsens to a critical stage [1]. In most cases, 95% of people survived to a certain stage, and the remaining 5% of people suffered from the advanced stage. It has also been observed that COVID-19 has affected more men than women, and children in the age group of 0–6 are at risk of infection. 

Since March 2020, several openly accessible X-ray images of COVID-19-infected persons have existed. It offers a method of analyzing the medical images and identifying every possible prototype that may lead to the automatic identification and classification of diseases. Presently, the imprints of this virus are stimulating effort because the unreachability of the COVID-19 diagnosis process results in stress globally. Because of the inadequate availability of COVID-19 rapid test kits, it has become essential to rely on other diagnostic techniques. Since the coronavirus damages the epithelial cells in the respiratory system, doctors make use of X-rays to diagnose the patient’s lungs [2]. As the hospitals commonly have X-ray imaging equipment, it becomes easy to test COVID-19 using X-rays without specific test kits. Radiological imaging techniques have become essential to detecting and classifying COVID-19. Although it denotes a circular allocation in the images, it exhibits identical characteristics to the alternative viral pandemic lung contagion. Because the coronavirus continues to grow quickly, different varieties of examinations are performed. 

Deep learning (DL) is an effective method involved in the healthcare-based diagnostic process. DL is a combination of machine learning (ML) algorithms and is majorly focused on automated feature extraction and classification processes [3,4]. ML as well as DL models have been recognized as well-identified models to mine, examine, and identify the patterns that exist in the images. Improving the progression of medical decision making and computer-aided design (CAD) turned out to be non-trivial, as effective data are produced [5]. DL, normally named deep CNN (DCNN), was utilized for automatically extracting the features that utilize the convolutional processes, and layers operate on nonlinear data. All the layers have a data transformation for superior and more abstract levels. Usually, DL refers to novel deep networks related to standard ML techniques utilizing big data [6].

This paper presents an automated glowworm swarm optimization (GSO) with an inception-based deep convolutional neural network (IDCNN) for COVID-19 diagnosis and classification, called the GSO-IDCNN model. The presented model utilizes a Gaussian smoothening filter (GSF) to exterminate the noise that occurs from the radiological images. Moreover, the IDCNN-based feature extractor was utilized, which employs the Inception v4 method. To further boost the performance of the IDCNN model, the hyperparameters are optimally tuned using the GSO algorithm. Finally, an adaptive neuro-fuzzy classifier (ANFC) is used for classifying the existence of COVID-19. For experimental validation, a series of simulations were performed on benchmark radiological imaging databases to highlight the superior outcome of the GSO-IDCNN model. In short, the contribution of the paper is listed as follows:

- To develop a new GSO-ODCNN model for COVID-19 detection and classification;
- To present a new GSF model to eradicate the noise that exists in the radiological images;
- To introduce a GSO model with an Inception v4-based feature extractor on radiological images;
- To employ ANFC classifier to allocate proper class labels to it;
- To validate the performance of the GSO-ODCNN model on the benchmark dataset.

## 2. Related Works

ML algorithms fall under the topic of artificial intelligence (AI), which is commonly employed for healthcare applications for feature extraction and image examination purposes. A classification model is developed for computing the dissimilarity amongst a collection of Regions of Interest (ROIs) [7,8]. Moreover, the features are classified by a normal vector-oriented classifier technique. Another *computed tomography* (CT)-based classification model is developed in [9] incorporating three classical features, such as grayscale values, shapes, textures, and symmetric features. It can be performed using RBFNN to classify the features involved in the images. A comparative study of JeffriesñMatusita (JñM) distance and KarhunenñLoËve transformation-based feature extracting techniques were developed [10].

A new classifier model is projected in [11] with an average grayscale value of images for a multi-class image classifier. A novel automatic classifier technique is developed in [12] for classifying breast cancer utilizing morphological features. Moreover, it is noticed that the outcome decreases when an identical process is carried out on an alternative dataset. Additionally, handcrafted techniques undergo initialization for deploying CNN and automated feature extraction methods. 

Ozyurt et al. [13] presented the hybridization technique known as fused perceptual hash dependent on the CNN model to decrease the diagnosis time of liver CT images and sustain the overall operation. Xu et al. [14] have executed a DTL technique to address the medicinal imaging imbalance problem. Lakshmanaprabu et al. [15] have investigated CT lung images using an optimum DNN as well as LDA. In [16], a transformation of original CT images to lower attenuation actual images and higher attenuation pattern rescaling was carried out. At last, the resampling of the images takes place and is classified using the CNN technique. A DL-based automatic lung and affected region segmentation process take place in [17] using a chest CT image. Wang et al. [18] relied on COVID-19 radiographical modifications in CT images and designed a DL model for graphical feature extraction of COVID-19, offering a medicinal examination prior to obtaining the pathogenic state to avert the deadly disorder in the patients. In [19], data mining (DM) techniques are applied to classifier SARS and pneumonia using X-rays. 

Although numerous techniques exist to diagnose COVID-19, it is still a requirement to analyze COVID-19 using chest X-ray images. X-ray machinery appeared to help scan the body for damage, such as fracture, bone displacement, lung disease, pneumonia, and tumor. By using X-rays, the scanning process is easy, quick, cheap, and harmless over CT. Since the advanced stage of COVID-19 leads to serious illnesses, a proficient CAD model for COVID-19 diagnosis is essential. At the same time, most of the earlier works have concentrated on binary classification. Therefore, in this study, a multi-label classification process is designed for COVID-19 diagnosis.

## 3. The Proposed GSO-IDCNN Model

**Figure 1:**
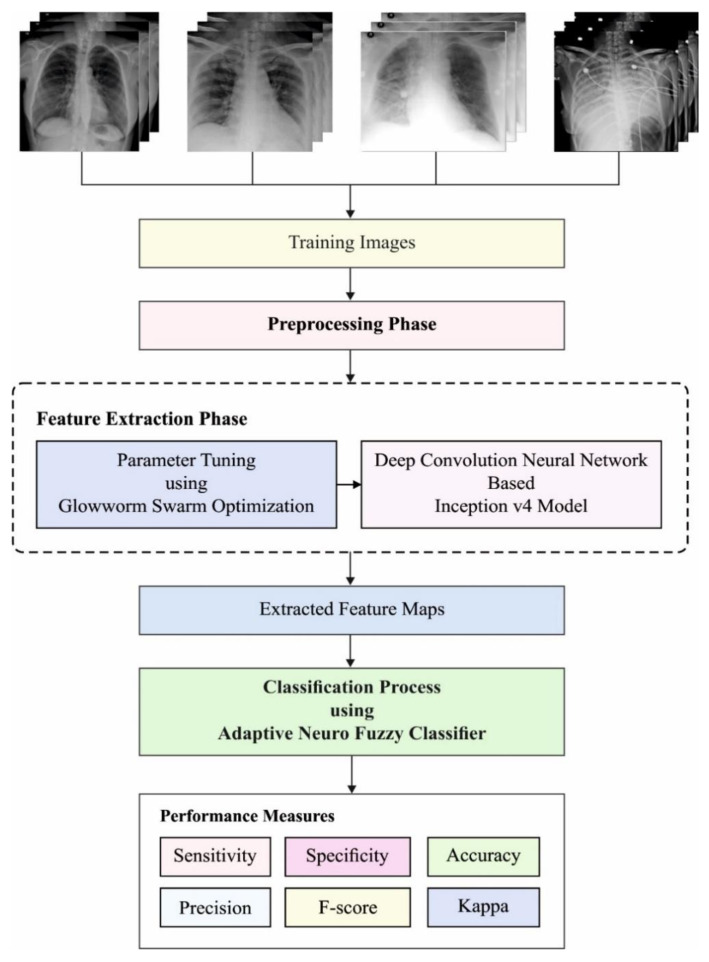
Working process of the GSO-IDCNN model.

The working procedure contained in the GSO-IDCNN technique is showcased in Figure 1. As depicted, the noise that exists from the radiological images is discarded by the GSF technique. Then, the feature extraction process takes place using the IDCNN model, where the parameters involved in it are tuned by the GSO technique. Eventually, the classification process is executed by the ANFC model to allocate appropriate class labels to it. 

### 3.1. GSF-Based Preprocessing

The design of 2D GSF is commonly employed to smoothen and remove noise. It necessitates massive computational time, and its effectiveness in the design is fascinating. 

The convolutional operators are the Gaussian operators, and the model of Gaussian smoothing is attained by convolutional operations. The 1D Gaussian operator has been represented by:

(1) $$G_{\mathrm{lD}}\left(x\right)=\frac{1}{\sqrt{2\pi }o}e^{-}\left(\frac{x^{2}}{2o^{2}}\right)\;$$

A better smoothening filtering process for images is recognized from the spatial and frequency domains, thus sustaining the uncertainty connection, as provided by [20]:

(2) $$\Delta x\Delta \omega \geq \frac{1}{2}.$$

The 2D Gaussian operator (circularly symmetric) can be represented by:

(3) $$G_{2D}\left(x,y\right)=\frac{1}{2\pi \sigma^{2}}e^{-}\left(\frac{x^{2}+y^{2}}{2\sigma^{2}}\right),\;$$

where σ designates the standard deviation (SD) of the Gaussian function. Once it includes a high value, the smoothening effect is found to be high, and (x, y) designates the Cartesian co-ordinate points in the image that indicates the window dimensional.

This filtering technique contains addition as well as multiplication tasks amongst the image and kernel. An image can be defined as a matrix with values of 0–255. The kernel was considered as a normalized square matrix that lies within the range of zero to one. The kernel can be defined using a specific bit count. 

The *MSE* is a cumulative square error amongst the reconstructed and original images that can be represented by:

(4) $$MSE=\frac{1}{M\times N}\overset{\;}{\underset{i}{\sum }}\overset{\;}{\underset{j}{\sum }}\left(O_{image}-R_{\mathrm{image}}\right),\;\;\;$$

where M×N indicates the image size, $O_{image}$ implies the original images, and $R_{image}$ denotes the restoring image. *PSNR* is the peak value of SNR, and it can be represented by the ratio of maximum probable power of pixel values and power of distorted noise. It affects the actual quality of the image and is represented by:

(5) $$PSNR=10\log_{10}\left[\frac{255\times 255}{MSE}\right],$$

where 255 × 255 is the higher pixel values that exist from the image, and *MSE* is determined to input and saved images with M×N size. The convolutional process is the multiplication method, and the informed logarithm product is ineffective with respect to the accurateness. Thus, it is an effectual logarithm multiplier for improving the accurateness of the Gaussian filter. 

### 3.2. IDCNN-Based Feature Extraction Model

**Figure 2:**
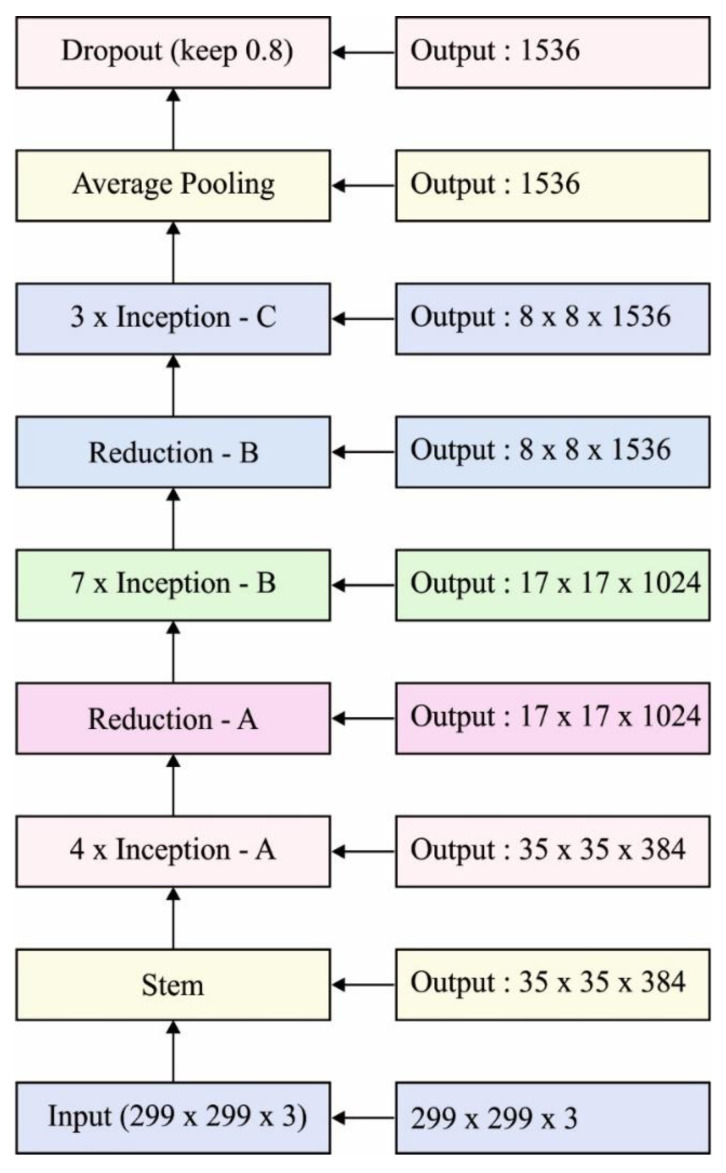
Network schema of Inception v4.

In this section, the features in the preprocessed image are filtered using the IDCNN-based Inception v4 model [21]. The older Inception versions are useful for training distinct blocks where all the repetitive blocks are split into a number of subnetworks enabling the total memory. However, the Inception network is easily tuned, demonstrating that several modifications are performed dependent upon the count of filters in different layers that do not control the quality of completely trained networks. To optimally elect the trained rate, the layer size needs to be set optimally to reach an effective tradeoff between processing and distinct subnetworks. Figure 2 illustrates the network schema of Inception v4. By contrast, in Tensor Flow, advanced Inception techniques are represented without any replica partition. 

For the residual version of the Inception network, the lower Inception blocks were obtainable on regular Inception. Every Inception block arrives in the filter-expansion layer, which increases the filtering bank’s dimensionality before the remaining summation to match the input depth. Further variation amongst the remaining and non-remaining Inception methods is that batch-normalization (BN) was applied on the conventional layer, then not on the peak value of the remaining summaries. It can be anticipated to the exclusive exploitation of BN is suitable, yet the plan of BN in TensorFlow necessitates massive memory, so it becomes essential for minimizing the layer count. Thus, BN is employed. 

It is expected that if the filter count exceeds 1000, the residual version starts offering uncertainty, and the network “dies” beforehand from the training, signifying that the final layer prior to average pooling creates only 0 s over different counts of iterations. Therefore, the minimization remaining prior to attaching the preceding activation layer is steady at the time of training. Usually, a few scaling factors exist in the interval of [0.1–0.3] to scale the residual prior to attaching it to the accumulated layer activation.

### 3.3. GSO-Based Hyperparameter Optimization Model

To optimize the hyperparameters of the GSO technique, a collection of glowworms is initialized and arbitrarily distributed from the solution space in such a way that is effective. The intensity of emitted lights was linked to the amount of luciferin that is closely integrated into it, whereas the glowworms were located from their motion and had a dynamic decision range $r_{d}^{i}\left(t\right)$ limited by a spherical sensor range $rs\;(0<r_{d}^{i}<=r_{s}^{i})$. Firstly, the glowworm comprises an identical count of luciferins, $l_{0}$. Based on the resemblance of luciferin values, the glowworm i selects their adjacent one j with probability $p_{ij}$ and shifts from the direction of decision ranges $rs(0<r_{d}^{i}<=r_{s}^{i})$, whereas the position of the glowworm i is represented by xi (xi∈Rm,i=1,2,…, n) [22].

A luciferin update stage is affected by the function value in the glowworms’ place. During the luciferin upgrade, the principle can be defined as:

(6) $$l_{i}\left(t+1\right)=\left(1-\rho \right)l_{i}\left(t\right)+\gamma J\left(x_{i}\left(x+1\right)\right)$$

where $l_{i}\left(t\right)$ denotes the luciferin level connected to a glowworm i at time t, ρ refers to the luciferin decay constant 0<ρ<1, γ represents the luciferin improvement constant, and $J\left(x_{i}\left(t\right)\right)$ signifies the value of the main function at agent i’s place at time t.

Along with the processes involved in the GSO technique, glowworms are fascinated by their neighbors that glow brighter. Thus, the outcome, at the time of the movement phase, the glowworms make use of the probabilistic process to move towards the neighbor that has a maximum luciferin intensity. In the case of every glowworm i, the possibility of moving over a neighboring glowworm can be represented as:

(7) $$p_{ij}\left(t\right)=\frac{l_{j}\left(t\right)-l_{j}\left(t\right)}{\Sigma_{k\in N_{i}\left(t\right)l_{k}\left(t\right)-l_{i}\left(t\right)}}$$

where $j\in N_{i}\left(t\right)$, $N_{i}\left(t\right)=\{j:d_{ij}\left(t\right)<r_{d}^{i}\left(t\right),\;l_{i}\left(t\right),\;l_{i}\left(t\right)<l_{j}\left(t\right)\}$ denotes the collection of nearby glowworms i at time t, $d_{ij}\left(t\right)$ indicates the Euclidean distance amongst the glowworm i and j at time t, and $r_{d}^{i}\left(t\right)$ denotes the variable neighboring range related to glowworm i at time t. The variable restricted by a radial sensor range ($0<r_{d}^{i}<rs$).

(8) $$x_{i}\left(t+1\right)=x_{i}\left(t\right)+s\left[\frac{x_{j}\left(t\right)-x_{i}\left(t\right)}{\left\|x_{j}\left(t\right)-x_{i}\left(t\right)\right\|}\right]$$

where s (>0) refers the step sizes, and ‖ ‖ implies the Euclidean norm operator. Moreover, $x_{i}\left(t\right)\in R^{m}$ denote the place of glowworm i at time t from the m dimensional real space $R^{m}$. Afterward, let $r_{0}$ be the initialized neighborhood ranges of all the glowworms (i.e, $r_{d}^{i}\left(0\right)=r_{0}$, ∀i):

(9) $$r_{d}^{i}\left(t+1\right)=\;\min\;\left\{r_{s},\;\max\;\left\{0,\;r_{d}^{i}\left(t\right)+\beta \left(n_{r}-\left|N_{i}\left(t\right)\right|\right)\right\}\right\}$$

where β is a constant, and $n_{t}$ defines a parameter utilized to control the degree. 

### 3.4. ANFC-Based Classification Model

**Figure 3:**
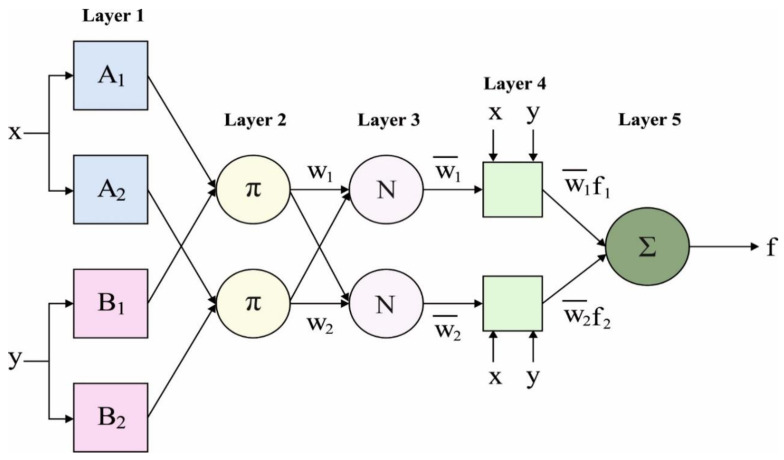
ANFC structure.

The ANFIS-based classification model can be employed to determine the class labels of the input radiological images. For simplicity, it is considered a network with two inputs, u and v, and one outcome, f. The ANFIS is a fuzzy Sugeno method. In order for the ANFIS structure to exist, two fuzzy if-then principles depend on the first-order Sugeno method, which is regarded as follows:

Rule 1: if u is A and v is $B_{1}$, then $f_{1}$ $=p_{1}u+q_{1}v+r_{1}$;
Rule 2: if u is A and v is $B_{2}$, then $f_{2}=p_{2}u+q_{2}v+r_{2}$;

where u and v are the input, A and $B_{i}$ are the fuzzified groups, $f_{i},$ i=1, 2 are the resultants of the fuzzy model, and $p_{i},$ $q_{i}$, and $r_{i}$ are the designing measures which is defined in the training model. The ANFIS structure for applying these two rules is demonstrated in Figure 3 [23], where the circle refers to the fixed node and the square denotes an adaptive node. As shown in the figure, the ANFIS structure has five layers.

Layer 1: All the nodes in layer 1 creates the adaptive node. The resultants of layer 1 are the fuzzified membership grade of input and are provided as:

(10) $$O_{i}^{1}={µ}_{A_{i}}\left(u\right)\;\;i=1,2\;\;\;\;\;\;$$

(11) $$O_{i}^{1}={µ}_{B_{i-2}}\left(u\right)\;\;i=3,4\;\;$$

where u and v are the inputs to node I, A refers the linguistic label, and ${µ}_{A_{i}}\left(u\right)$ and ${µ}_{B_{i-2}}\left(u\right)$ accept some fuzzy membership function (MF). In general, $\propto_{A_{i}}\left(u\right)$ is chosen by:

(12) $${µ}_{A_{i}}=\frac{1}{1+{\left\{{\left[\left(u-c_{i}\right)/a_{i}\right]}^{2}\right\}}^{b_{i}}}\;\;\;\;\;\;$$

where $a_{i},$ $b_{i}$, and $c_{i}$ are the measures of membership bell-shaped functions.

Layer 2: A node in this layer is labeled M, reflecting that it is executed by a simple multiplier. The resultants of the layer are illustrated as:

(13) $$O_{i}^{2}=w_{i}={µ}_{A_{i}}\left(u\right){µ}_{B_{i}}\left(v\right)i=1,2\;\;\;\;\;\;\;$$

Layer 3: It has static nodes that compute the ratio of firing strength of the principles, as given below:

(14) $$O_{i}^{3}={\bar{w}}_{i}=\frac{w_{i}}{w_{1}+w_{2}}\;i=1,2\;\;\;\;\;\;\;\;\;\;\;\;\;$$

Layer 4: In this layer, nodes are adaptive nodes. The resultants of this layer are calculated by the procedure provided below:

(15) $$O_{i}^{4}={\bar{w}}_{i}f_{i}={\bar{w}}_{i}\left(p_{i}u+q_{j}v+r_{i}\right)\;i=1,2\;\;\;$$

where ${\bar{w}}_{i}$ is a normalized firing strength from layer 3.

Layer 5: A node executes the summary of each received signal. Therefore, an entire output of the method is provided as:

(16) $$O_{i}^{5}=\underset{i}{\sum }{\bar{w}}_{i}f_{i}=\frac{\sum_{i}w_{i}f_{i}}{\sum_{i}w_{i}}\;\;\;$$

There are two adaptive layers in this ANFIS model, i.e., the first and fourth layers. In the first layer, there are three modifiable measures $\left\{a,\;b_{i},c_{i}\right\}$ that are compared with the input MFs. These measures are usually known as premise measures. The fourth layer is also three modifiable measures $\left\{p_{i}\;q_{i}\;r_{i}\right\}$ relating to the first-order polynomial. The consequent measures are during this measurement [24]. 

### 3.5. Learning Algorithm of ANFIS

The learning technique for this model is to tune every modifiable measure, such as $\left\{a_{i}b_{i}c_{i}\right\}$ and $\left\{p_{i}q_{i}r_{i}\right\}$, to create an ANFIS output matching the trained data. If premise measures $a_{i}$*,* $b_{i}$*,* and $c_{i}$ of the MFs are suitable, the resultant of the ANFIS method is expressed by:

(17) $$f=\frac{w_{1}}{w_{1}+w_{2}}f_{1}+\frac{w_{2}}{w_{1}+w_{2}}f_{2}\;\;\;\;$$

By replacing Equation (14) and fuzzy if-then principles with Equation (8), it develops:

(18) $$f={\bar{w}}_{1}\left(p_{1}+q_{1}v+r_{1}\right)+{\bar{w}}_{2}\left(p_{2}u+q_{2}v+r_{2}\right)\;\;\;$$

where ${\bar{w}}_{1},{\bar{w}}_{2}$ are calculated by Equation (14). After the rearrangement, the output is demonstrated by:

(19) $$f=\left({\bar{w}}_{1}u\right)p_{1}+\left({\bar{w}}_{1}v\right)q_{1}+\left({\bar{w}}_{1}\right)r_{1}+\left({\bar{w}}_{2}u\right)p_{2}+\left({\bar{w}}_{2}v\right)q_{2}+\left({\bar{w}}_{2}\right)r_{2}\;\;\;$$

with the linear grouping of changeable resultant measures $p_{1},$ $q_{1},$ $r_{1},$ $p_{2},$ $q_{2}$*,* and $r_{2}$. These measures are upgraded to forward pass the learning technique using the least squares model. Let q be an unidentified vector comprising six measures. Thus, Equation (19) is illustrated by:

(20) f=θA 

When *A* is an invertible matrix then:

(21) $$\theta =A^{-1}f\;\;\;$$

Then, a pseudo-inverse is utilized to solve q as follows:

(22) $$\theta ={\left(A^{T}A\right)}^{-1}A^{T}f\;\;\;\;$$

During the backward pass, the error signals are propagated, and premise measures are upgraded with gradient descent.

(23) $$\alpha_{new}=\alpha_{old}-\eta \;\frac{\partial E}{\partial \alpha }\;$$

where E is the MSE, α is all the premise measures, and η is the rate of learning. The chain rule is applied to calculate the partial derivative utilized for upgrading the MF measures.

(24) $$\frac{\partial E}{\partial \alpha }=\frac{\partial E}{\partial f}\frac{\partial f}{\partial f_{j}}\frac{\partial f_{j}}{\partial w_{j}}\frac{\partial w_{j}}{\partial \mu_{i}}\frac{\partial \mu_{i}}{\partial \alpha }\;$$

By following the above expression and computing all the partial derivatives, the premise measures $\left\{a_{i}b_{i}c_{i}\right\}$ are upgraded in Equation (23).

## 4. Experimental Validation

**Figure 4:**
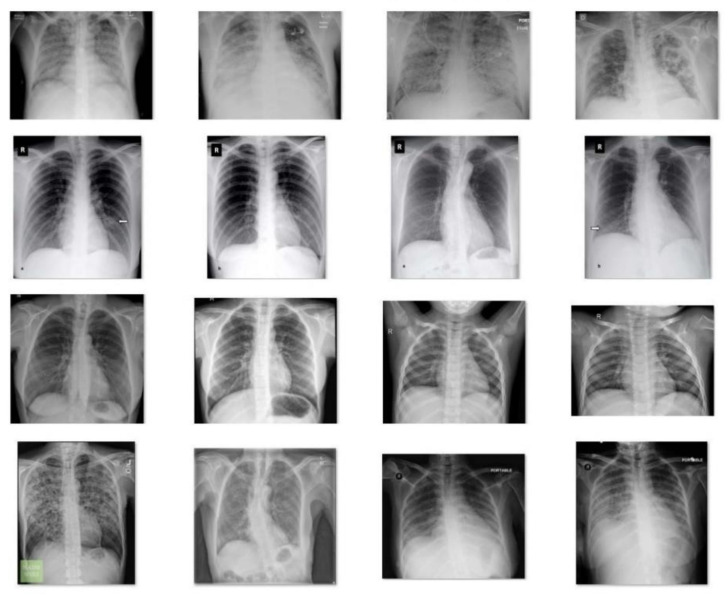
Sample Images.

To ensure the classification performance of the GSO-IDCNN method, an extensive experimental validation process was carried out with a chest X-ray dataset [25]. It encompasses a set of 220 COVID-19 images, 27 normal images, 15 pneumocystis images, and 11 SARS images. Figure 4 showcases the sample images. The presented method was executed using an Intel i5, 8th-generation PC with 16GB RAM, MSI L370 Apro, Nividia 1050 Ti4 GB. For experimentation, the Python 3.6.5 tool was utilized together with Pillow, pandas, sklearn, TensorFlow, Keras, opencv, seaborn, Matplotlib, and pycm. The parameters contained are batch size: 128, learning rate: 0.001, epoch count: 500, and momentum: 0.2. 

**Figure 5:**
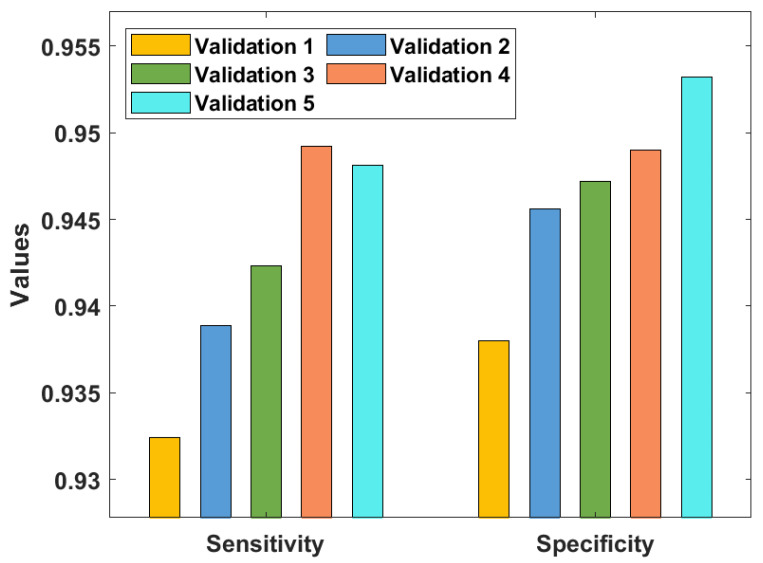
Result analysis of the GSO-IDCNN approach with respect to $sens_{y}$ and $spec_{y}$.

**Figure 6:**
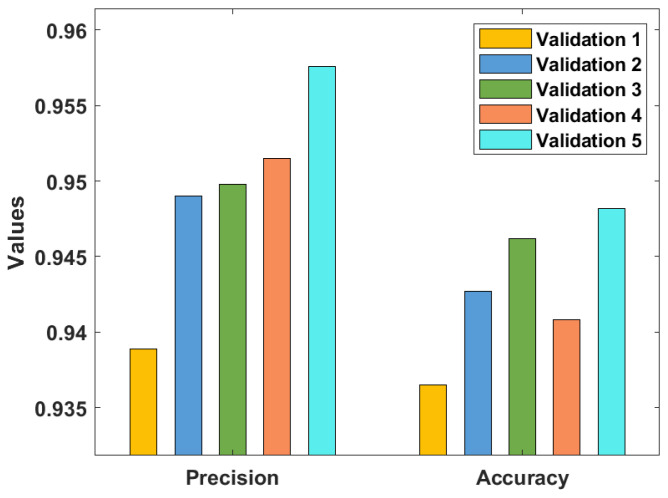
Result analysis of the GSO-IDCNN technique with respect to $Prec_{n}$ and $Acc_{y}$.

**Figure 7:**
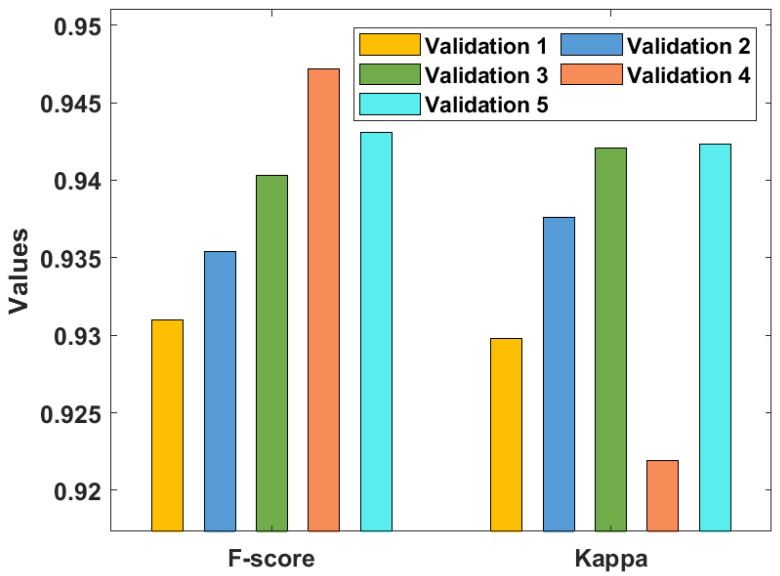
Result analysis of the GSO-IDCNN technique with respect to $F1_{score}$ and kappa.

Table 1 and Figure 5, Figure 6 and Figure 7 investigate the classifier outcome analysis of the GSO-IDCNN model under several kinds of validation. The GSO-IDCNN model obtained effective diagnostic outcomes by offering higher performance. For the samples on validation 1, the GSO-IDCNN approach has reached higher $sens_{y}$, $spec_{y}$, $prec_{n}$, $acc_{y}$, $F1_{score}$, and kappa values of 0.9324, 0.9380, 0.9389, 0.9365, 0.9310, and 0.9298, respectively. 

Eventually, in validation 2, the GSO-IDCNN method attained superior $sens_{y}$, $spec_{y}$, $prec_{n}$, $acc_{y}$, $F1_{score}$, and kappa values of 0.9389, 0.9456, 0.9490, 0.9427, 0.9354, and 0.9376, respectively. Moreover, in validation 3, the GSO-IDCNN approach gained increased $sens_{y}$, $spec_{y}$, $prec_{n}$, $acc_{y}$, $F1_{score}$, and kappa values of 0.9423, 0.9472, 0.9498, 0.9462, 0.9403, and 0.9421, respectively. Further, in validation 4, the GSO-IDCNN model gained maximal $sens_{y}$, $spec_{y}$, $prec_{n}$, $acc_{y}$, $F1_{score}$, and kappa values of 0.9492, 0.9490, 0.9515, 0.9408, 0.9472, and 0.9219, respectively. Furthermore, in validation 5, the GSO-IDCNN method achieved superior sensitivity, specificity, precision, accuracy, F1-score, and kappa values of 0.9481, 0.9532, 0.9576, 0.9482, 0.9431, and 0.9423, respectively.

**Figure 8:**
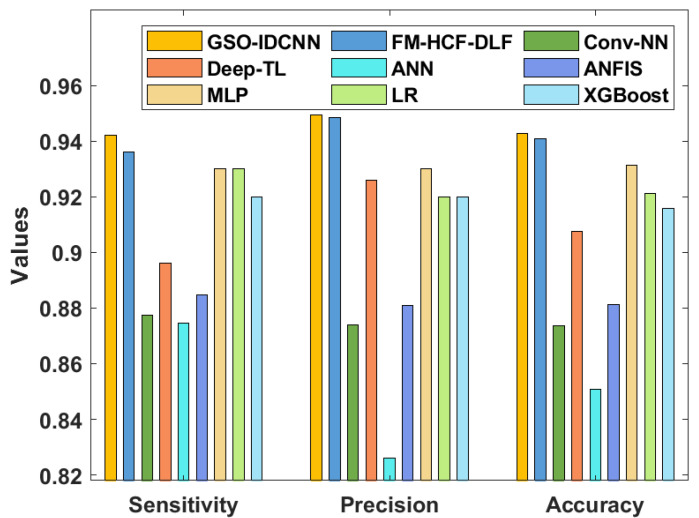
Comparative analysis of the GSO-IDCNN technique with different measures.

**Figure 9:**
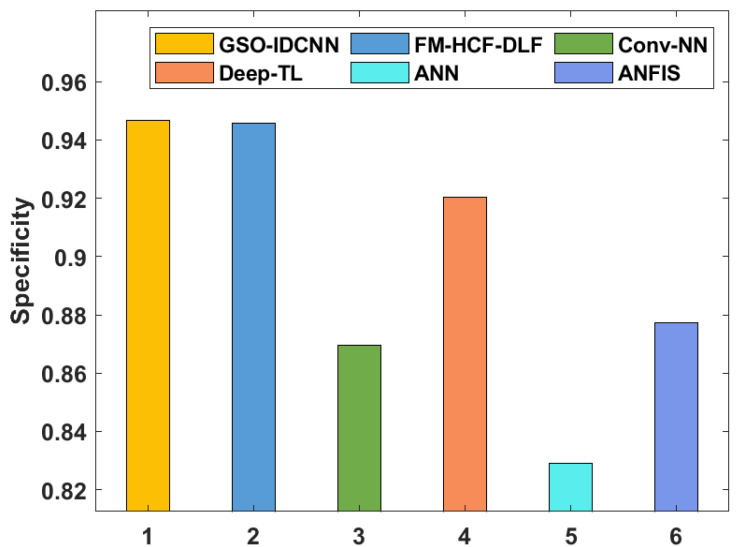
Comparative analysis of the GSO-IDCNN technique with respect to $spec_{y}$.

Table 2 and Figure 8 and Figure 9 offer a detailed comparative analysis of the GSO-IDCNN technique with respect to distinct measures [26]. The $sens_{y}$ analysis of the GSO-IDCNN approach with existing algorithms displays that the ANN approach accomplished ineffective results with a lower $sens_{y}$ value of 0.8745. Moreover, the Conv-NN system resulted in a somewhat increased $sens_{y}$ value of 0.8773, whereas the ANFIS and Deep-TL models have accomplished reasonably closer $sens_{y}$ values of 0.8848 and 0.8961, respectively. Eventually, the XGBoost algorithm demonstrated a reasonable outcome with a $sens_{y}$ value of 0.92. Afterward, the MLP and LR approaches depicted considerably increased $sens_{y}$ values of 0.93 and 0.93. Though the FM-HCF-DLF methodology offered a slightly better $sens_{y}$ value of 0.9361, the presented GSO-IDCNN technique achieved a maximum $sens_{y}$ value of 0.9422.

The $spec_{y}$ analysis of the GSO-IDCNN approach with recent methodologies demonstrates that the ANN model accomplished ineffective outcomes with the minimal $spec_{y}$ value of 0.8291. Additionally, the Conv-NN system resulted in a somewhat increased $spec_{y}$ value of 0.8697, whereas the ANFIS model accomplished a moderate $spec_{y}$ value of 0.8774. Next, the Deep-TL approach showcased reasonable outcomes with a $spec_{y}$ value of 0.9203. Afterward, the FM-HCF-DLF model depicted a considerably increased $spec_{y}$ value of 0.9456. However, the proposed GSO-IDCNN system gained a superior $spec_{y}$ value of 0.9466.

The $prec_{n}$ analysis of the GSO-IDCNN technique with the existing methods shows that the ANN methodology accomplished an ineffectual outcome with a minimum $prec_{n}$ value of 0.8259. In line with this, the Conv-NN model resulted in a slightly higher $prec_{n}$ value of 0.8741, whereas the ANFIS approach accomplished a moderate $prec_{n}$ value of 0.8808. Similarly, the LR and XGBoost models demonstrated a similar $prec_{n}$ value of 0.9200. In addition, the Deep-TL approach portrayed a reasonable outcome with a $prec_{n}$ value of 0.9259. Next, the MLP model has depicted a considerably increased $prec_{n}$ value of 0.9300. Although the FM-HCF-DLF methodology offered a slightly better $prec_{n}$ value of 0.9485, the proposed GSO-IDCNN model achieved a higher $prec_{n}$ value of 0.9494.

The $acc_{y}$ analysis of the GSO-IDCNN methodology with existing approaches exhibits that the ANN method has accomplished ineffective results with a minimum $acc_{y}$ of 0.8509. Similarly, the Conv-NN model resulted in a somewhat enhanced $acc_{y}$ of 0.8736, whereas the ANFIS and Deep-TL systems accomplished reasonably closer $acc_{y}$ values of 0.8811 and 0.9075, respectively. Following them, the XGBoost approach illustrated a reasonable outcome with an $acc_{y}$ value of 0.9157. Concurrently, the LR and MLP methodologies depicted considerably improved $acc_{y}$ values of 0.9212 and 0.9313. Although the FM-HCF-DLF model offered a near-optimal $acc_{y}$ of 0.9408, the projected GSO-IDCNN technique reached a superior $acc_{y}$ of 0.9429. Finally, the $F1_{score}$ analysis of the GSO-IDCNN approach with the existing methodologies displays that the LR and XGBoost methods accomplished ineffective results with the smallest $F1_{score}$ of 0.9200. Additionally, the MLP system resulted in a somewhat maximum $F1_{score}$ of 0.9300. Eventually, the FM-HCF-DLF model outperformed the reasonable results with an $F1_{score}$ of 0.9320. However, the proposed GSO-IDCNN methodology achieved a superior $F1_{score}$ of 0.9394.

From the brief experimental validation, we have ensured that the GSO-IDCNN technique exhibited an effective diagnostic performance on the related approaches since it provided a maximal $sens_{y}$ value of 0.9422, a $spec_{y}$ value of 0.9466, a $prec_{n}$ value of 0.9494, an $acc_{y}$ value of 0.9429, and an $F1_{score}$ of 0.9394. It is due to the integration of the SSA for the parameter tuning of IDCNN using GSO and ANFC models.

## 5. Conclusions

This paper has established a GSO-IDCNN approach for COVID-19 diagnosis and classification. Primarily, the noise that occurs from radiological images is discarded by the GSF technique. Then, the feature extraction process occurs utilizing the IDCNN model, where the parameters involved in it are tuned by the GSO technique. Eventually, the classification process is executed by the ANFC model to allocate appropriate class labels to it. To validate the performance of the GSO-IDCNN method, extensive simulation analyses were carried out on the benchmark radiological imaging databases to highlight the superior outcome of the GSO-IDCNN technique. The experimental values pointed out that the GSO-IDCNN approach has demonstrated proficient outcome by offering a maximal $sens_{y}$ value of 0.9422, a $spec_{y}$ value of 0.9466, a $prec_{n}$ value of 0.9494, an $acc_{y}$ value of 0.9429, and an $F1_{score}$ of 0.9394. In the future, the COVID-19 diagnostic performance could be improved by utilizing advanced end-to-end deep learning architectures.

## Figures and Tables

**Table 1:** Result analysis of the presented GSO-IDCNN technique with respect to distinct measures.

No. of Validation	$Sens_{y}$	$Spec_{y}$	$Prec_{n}$	$Acc_{y}$	$F1_{score}$	Kappa
Validation 1	0.9324	0.9380	0.9389	0.9365	0.9310	0.9298
Validation 2	0.9389	0.9456	0.9490	0.9427	0.9354	0.9376
Validation 3	0.9423	0.9472	0.9498	0.9462	0.9403	0.9421
Validation 4	0.9492	0.9490	0.9515	0.9408	0.9472	0.9219
Validation 5	0.9481	0.9532	0.9576	0.9482	0.9431	0.9423
Average	0.9422	0.9466	0.9494	0.9429	0.9394	0.9347

**Table 2:** Comparative studies of the existing models with the presented GSO-IDCNN models.

Methods	$Sens_{y}$	$Spec_{y}$	$Prec_{n}$	$Acc_{y}$	$F1_{score}$
GSO-IDCNN	0.9422	0.9466	0.9494	0.9429	0.9394
FM-HCF-DLF	0.9361	0.9456	0.9485	0.9408	0.9320
Conv-NN	0.8773	0.8697	0.8741	0.8736	-
Deep-TL	0.8961	0.9203	0.9259	0.9075	-
ANN	0.8745	0.8291	0.8259	0.8509	-
ANFIS	0.8848	0.8774	0.8808	0.8811	-
MLP	0.9300	-	0.9300	0.9313	0.9300
LR	0.9300	-	0.9200	0.9212	0.9200
XGBoost	0.9200	-	0.9200	0.9157	0.9200

## Data Availability

Data sharing is not applicable to this article as no datasets were generated in the study.
